# SOFA in sepsis: with or without GCS

**DOI:** 10.1186/s40001-024-01849-w

**Published:** 2024-05-24

**Authors:** Lu Wang, Xudong Ma, Guanghua Zhou, Sifa Gao, Wei Pan, Jieqing Chen, Longxiang Su, Huaiwu He, Yun Long, Zhi Yin, Ting Shu, Xiang Zhou, China National Critical Care Quality Control Centre Group (China-NCCQC), Yongjun Liu, Yan Kang, Jing Yan, Erzhen Chen, Bin Xiong, Bingyu Qin, Kejian Qian, Wei Fang, Mingyan Zhao, Xiaochun Ma, Xiangyou Yu, Jiandong Lin, Yi Yang, Feng Shen, Shusheng Li, Lina Zhang, Weidong Wu, Meili Duan, Linjun Wan, Xiaojun Yang, Jian Liu, Zhen Wang, Lei Xu, Zhenjie Hu, Congshan Yang

**Affiliations:** 1 Department of Critical Care Medicine, State Key Laboratory of Complex Severe and Rare Diseases, Peking Union Medical College and Chinese Academy of Medical Sciences, Peking Union Medical College Hospital, Beijing, 100730 China; 2 Department of Medical Administration, National Health Commission of the People’s Republic of China, Beijing, 100044 China; 3 Department of Information Technology, Center of Statistics and Health Informatics, National Health Commission of the People’s Republic of China, Beijing, 100044 China; 4 Information Center Department/Department of Information Management, Peking Union Medical College Hospital, Peking Union Medical College and Chinese Academy of Medical Sciences, Beijing, 100730 China; 5 Department of Intensive Care Unit, The People’s Hospital of Zizhong, Neijiang, 641000 Sichuang China; 6 National Institute of Hospital Administration, Beijing, 100730 China

**Keywords:** GCS, SOFA, Sepsis, Central nervous system, Respiratory

## Abstract

**Purpose:**

Sepsis is a global public health burden. The sequential organ failure assessment (SOFA) is the most commonly used scoring system for diagnosing sepsis and assessing severity. Due to the widespread use of endotracheal intubation and sedative medications in sepsis, the accuracy of the Glasgow Coma Score (GCS) is the lowest in SOFA. We designed this multicenter, cross-sectional study to investigate the predictive efficiency of SOFA with or without GCS on ICU mortality in patients with sepsis.

**Methods:**

First, 3048 patients with sepsis admitted to Peking Union Medical College Hospital (PUMCH) were enrolled in this survey. The data were collected from June 8, 2013 to October 12, 2022. Second, 18,108 patients with sepsis in the eICU database were enrolled. Third, 2397 septic patients with respiratory system ≥ 3 points in SOFA in the eICU database were included. We investigated the predictive efficiency of SOFA with or without GCS on ICU mortality in patients with sepsis in various ICUs of PUMCH, and then we validated the results in the eICU database.

**Main results:**

In data of ICUs in PUMCH, the predictive efficiency of SOFA without GCS (AUROC [95% CI], 24 h, 0.724 [0.688, 0.760], 48 h, 0.734 [0.699, 0.769], 72 h, 0.748 [0.713, 0.783], 168 h, 0.781 [0.747, 0.815]) was higher than that of SOFA with GCS (AUROC [95% CI], 24 h, 0.708 [0.672, 0.744], 48 h, 0.721 [0.685, 0.757], 72 h, 0.735 [0.700, 0.757], 168 h, 0.770 [0.736, 0.804]) on ICU mortality in patients with sepsis, and the difference was statistically significant (P value, 24 h, 0.001, 48 h, 0.003, 72 h, 0.004, 168 h, 0.005). In septic patients with respiratory system ≥ 3 points in SOFA in the eICU database, although the difference was not statistically significant (P value, 24 h, 0.148, 48 h, 0.178, 72 h, 0.132, 168 h, 0.790), SOFA without GCS (AUROC [95% CI], 24 h, 0.601 [0.576, 0.626], 48 h, 0.625 [0.601, 0.649], 72 h, 0.639 [0.615, 0.663], 168 h, 0.653 [0.629, 0.677]) had a higher predictive efficiency on ICU mortality than SOFA with GCS (AUROC [95% CI], 24 h, 0.591 [0.566, 0.616], 48 h, 0.616 [0.592, 0.640], 72 h, 0.628 [0.604, 0.652], 168 h, 0.651 [0.627, 0.675]).

**Conclusions:**

In severe sepsis, it is realistic and feasible to discontinue the routine GCS for SOFA in patients with a respiratory system ≥ 3 points, and even better predict ICU mortality.

**Supplementary Information:**

The online version contains supplementary material available at 10.1186/s40001-024-01849-w.

## Key messages

- SOFA is the most commonly used scoring system for diagnosing sepsis and assessing severity. Due to the widespread use of endotracheal intubation and sedative medications in sepsis, the accuracy of GCS is the lowest in SOFA. We designed this multicenter, cross-sectional study to investigate the predictive efficiency of SOFA with or without GCS on ICU mortality in patients with sepsis.
- In severe sepsis, it is realistic and feasible to discontinue the routine GCS for SOFA in patients with a respiratory system ≥ 3 points, and even better predict ICU mortality.

**Supplementary Information:**

The online version contains supplementary material available at 10.1186/s40001-024-01849-w.

## Introduction

Sepsis is organ dysfunction due to severe infection and is one of the leading causes of death and critical illness worldwide [1, 2]. Without timely and effective intervention, mortality of sepsis can rapidly exceed 30–35% [3]. The sequential organ failure assessment (SOFA) is the most commonly used scoring system for diagnosing sepsis and assessing severity [4–6]. Due to the widespread use of endotracheal intubation and sedative medications in sepsis, the use of best guess methods or the continuation of pre-intubation recordings may overestimate the central nervous system function, thus affecting the prediction efficiency of SOFA [7]. In a study on the effects of levosimendan on acute organ dysfunction in sepsis, SOFA that does not include Glasgow Coma Score (GCS) was tried, and there was no significant decrease in evaluation efficiency [8]. With the above in mind, we designed this study to investigate the predictive efficiency of SOFA with or without GCS on ICU mortality in patients with sepsis in various ICUs of Peking Union Medical College Hospital (PUMCH), and then we validated the results in the eICU database.

## Methods

### Study design

This was an observational, retrospective study. In this survey, 3048 patients with sepsis admitted to PUMCH were enrolled. The data were collected from June 8, 2013 to October 12, 2022. The basic information of patients with sepsis in PUMCH were shown in Table 1. The eICU Database is a freely available multi-center database for critical care research. In the eICU database, 18,108 patients with sepsis were selected as validation set. The basic information of patients with sepsis in the eICU database were shown in Additional file 3: Table S1. Sepsis was diagnosed on the basis of the third international consensus definitions for sepsis and septic shock. Patient inclusion and exclusion criteria were provided in the Additional file 2: Fig. S1.

**Table 1:** Basic information of patients with sepsis in Peking Union Medical College Hospital (PUMCH)

	Patients (n)	Patients died in ICU (n)	ICU mortality (%)
Total	3048	422	13.85
Female	1201	174	14.49
Age (year)	61.0 (48.0, 69.0)	64.5 (53.0, 73.0)	
Weight (kg)	66.0 (58.0, 75.0)	65.0(57.0, 72.0)	
Chronic cardiovascular disease	2370	352	14.85
Chronic reapiratory diseases	1403	358	25.52
Chronic neurological diseases	432	96	22.22
Chronic kidney disease	1244	270	21.70
Chronic digestive diseases	1520	292	19.21
Diabetes mellitus	1337	281	21.02
Hematological cancer	166	65	39.16
Nonhematological cancer	768	103	13.41

The authors are accountable for all aspects of the work in ensuring that questions related to the accuracy or integrity of any part of the work are appropriately investigated and resolved. The datasets supporting the conclusions of this article are included within the article (see Additional file 1).

### Variables and measurements

Patients included in this study had completed SOFA during their ICU period. SOFA and GCS were completed by ICU nurses who were trained and qualified in critical care medicine. In this study, the best guess method based on clinical experience was used for GCS in patients receiving sedation. SOFA and GCS were performed within 24 h, 48 h, 72 h, and 168 h respectively and the worst results during the observation period were collected.

We first investigated the predictive efficiency of SOFA with or without GCS on ICU mortality in patients with sepsis in various ICUs of PUMCH, and then we validated the results in the eICU database.

### Ethical considerations

The current study was reported in accordance with the Strengthening the Reporting of Observational Studies in Epidemiology Guidelines. This study was conducted in accordance with the Declaration of Helsinki (as revised in 2013). The trial protocol was approved by the Central Institutional Review Board at Peking Union Medical College Hospital (NO. SK1828), and individual consent for this analysis was waived. There was no identifying or protected health information included in the analyzed dataset.

### Data analysis

All statistical analyses were performed in SAS 9.4 (SAS Institute Inc., Cary, NC, USA). Continuous variables were expressed as media (P25, P75). The area under the receiver operating characteristic curves (AUROCs) were used to evaluate the performance of variables at different time scales to predict ICU mortality. DeLong’s test was used to compare the differences in AUROCs. All p values were 2-tailed, and a p-value < 0.05 was considered statistically significant.

## Results

**Fig. 1:**
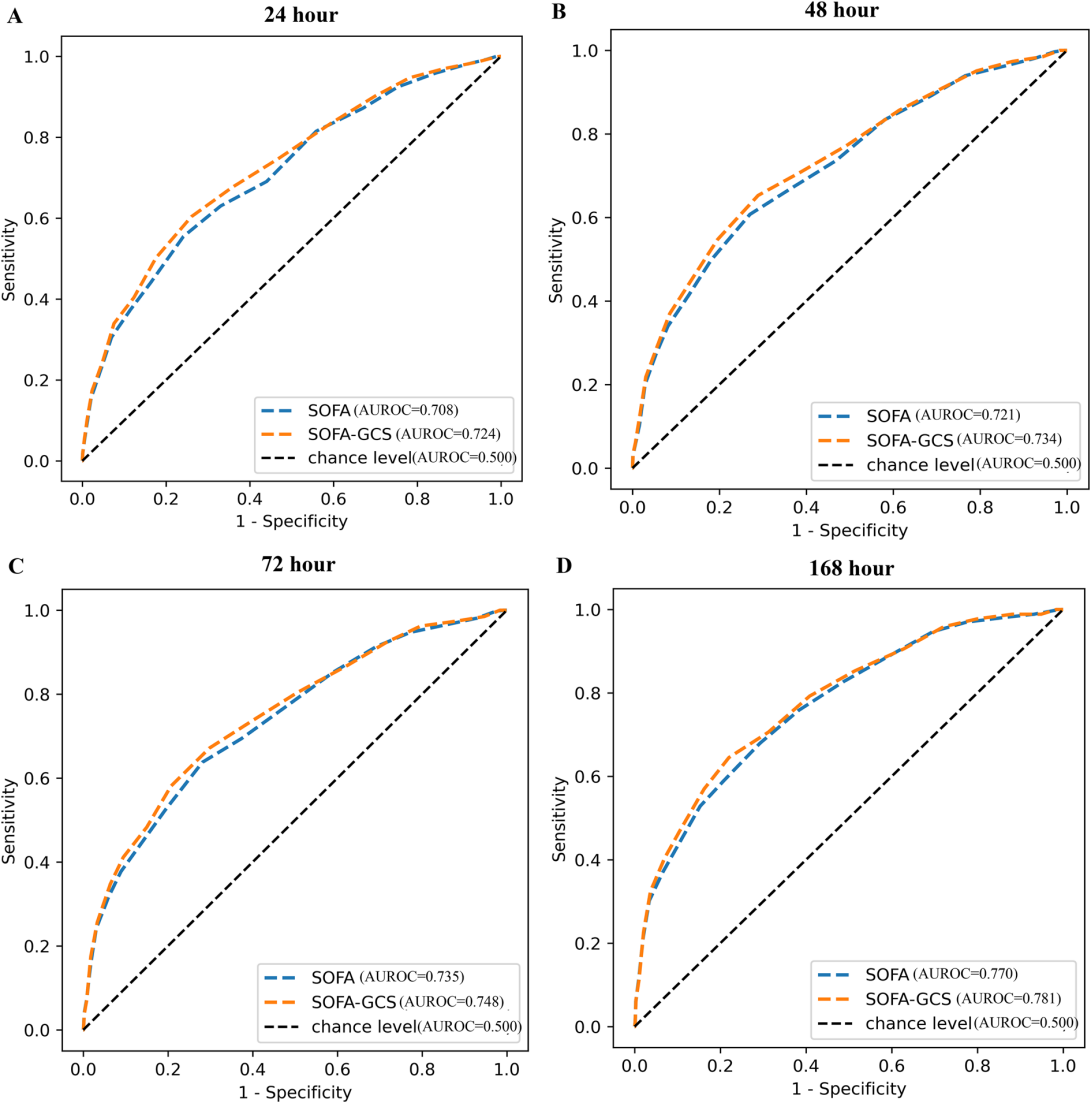
Predictive efficiency of SOFA with or without GCS in patients with sepsis in Peking Union Medical College Hospital (PUMCH). **A** p (SOFA vs SOFA-GCS) 0.001, **B** p (SOFA vs SOFA-GCS) 0.003, **C** p (SOFA vs SOFA-GCS) 0.004, **D** p (SOFA vs SOFA-GCS) 0.005

In data of ICUs in PUMCH, the predictive efficiency of SOFA without GCS (AUROC [95% CI], 24 h, 0.724 [0.688, 0.760], 48 h, 0.734 [0.699, 0.769], 72 h, 0.748 [0.713, 0.783], 168 h, 0.781 [0.747, 0.815]) was higher than that of SOFA with GCS (AUROC [95% CI], 24 h, 0.708 [0.672, 0.744], 48 h, 0.721 [0.685, 0.757], 72 h, 0.735 [0.700, 0.757], 168 h, 0.770 [0.736, 0.804]) on ICU mortality in patients with sepsis, and the difference was statistically significant (P value, 24 h, 0.001, 48 h, 0.003, 72 h, 0.004, 168 h, 0.005) (Fig. 1).

**Fig. 2:**
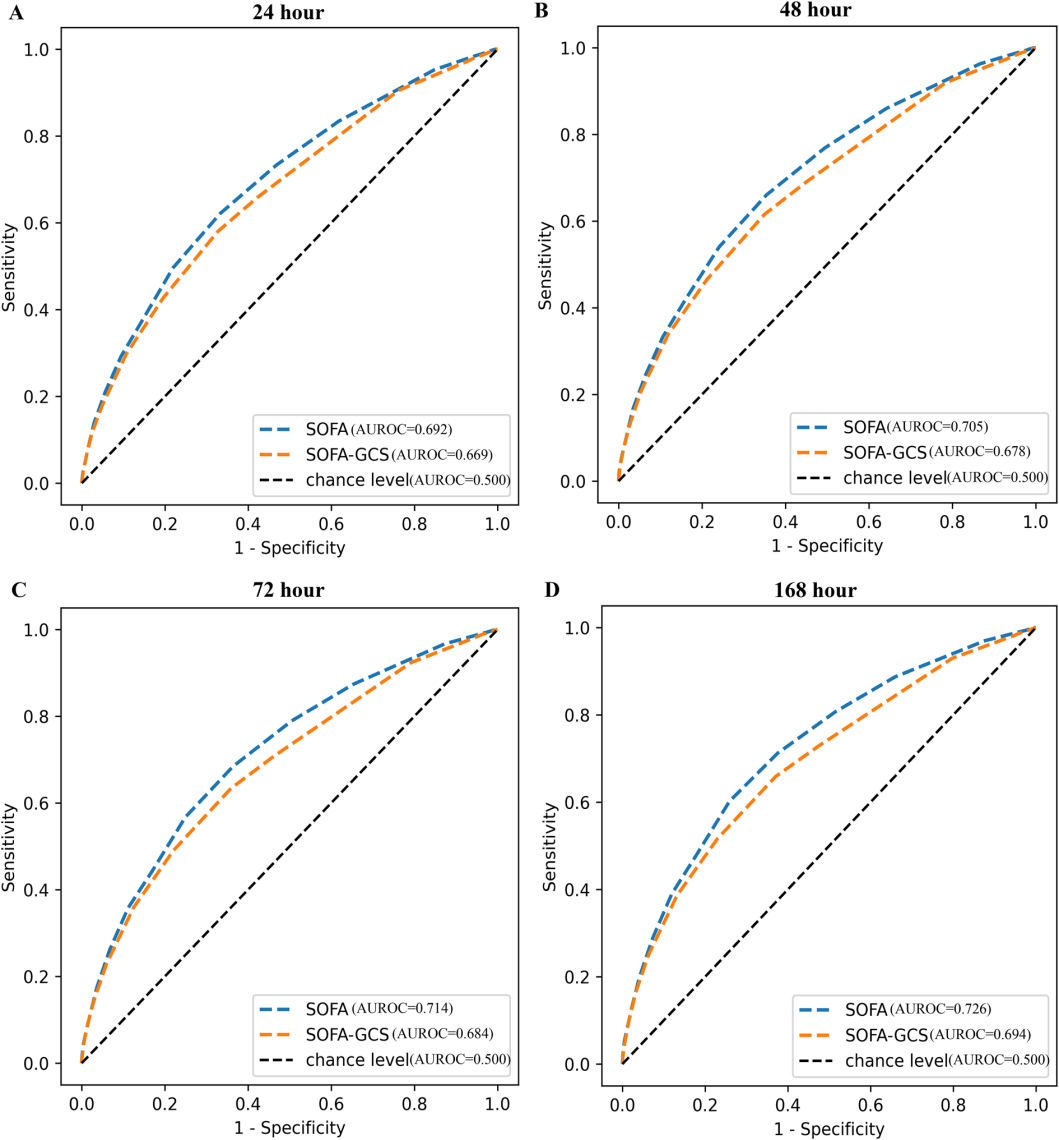
Predictive efficiency of SOFA with or without GCS in patients with sepsis in the eICU database. **A** p (SOFA vs SOFA-GCS) < 0.001, **B** p (SOFA vs SOFA-GCS) < 0.001, **C** p (SOFA vs SOFA-GCS) < 0.001, **D** p (SOFA vs SOFA-GCS) < 0.001

We tried to validate this result in the eICU database. A total of 18,108 patients with sepsis were included. However, no identical results were observed. In eICU database, the predictive efficiency of SOFA without GCS (AUROC [95% CI], 24 h, 0.669 [0.657, 0.681], 48 h, 0.678 [0.666, 0.690], 72 h, 0.684 [0.673, 0.695], 168 h, 0.694 [0.683, 0.705]) was lower than that of SOFA with GCS (AUROC [95% CI], 24 h, 0.692 [0.681, 0.703], 48 h, 0.705 [0.694, 0.716], 72 h, 0.714 [0.703, 0.725], 168 h, 0.726 [0.715, 0.737]) on ICU mortality in patients with sepsis (P value, 24 h, < 0.001, 48 h, < 0.001, 72 h, < 0.001, 168 h, < 0.001) (Fig. 2).

**Fig. 3:**
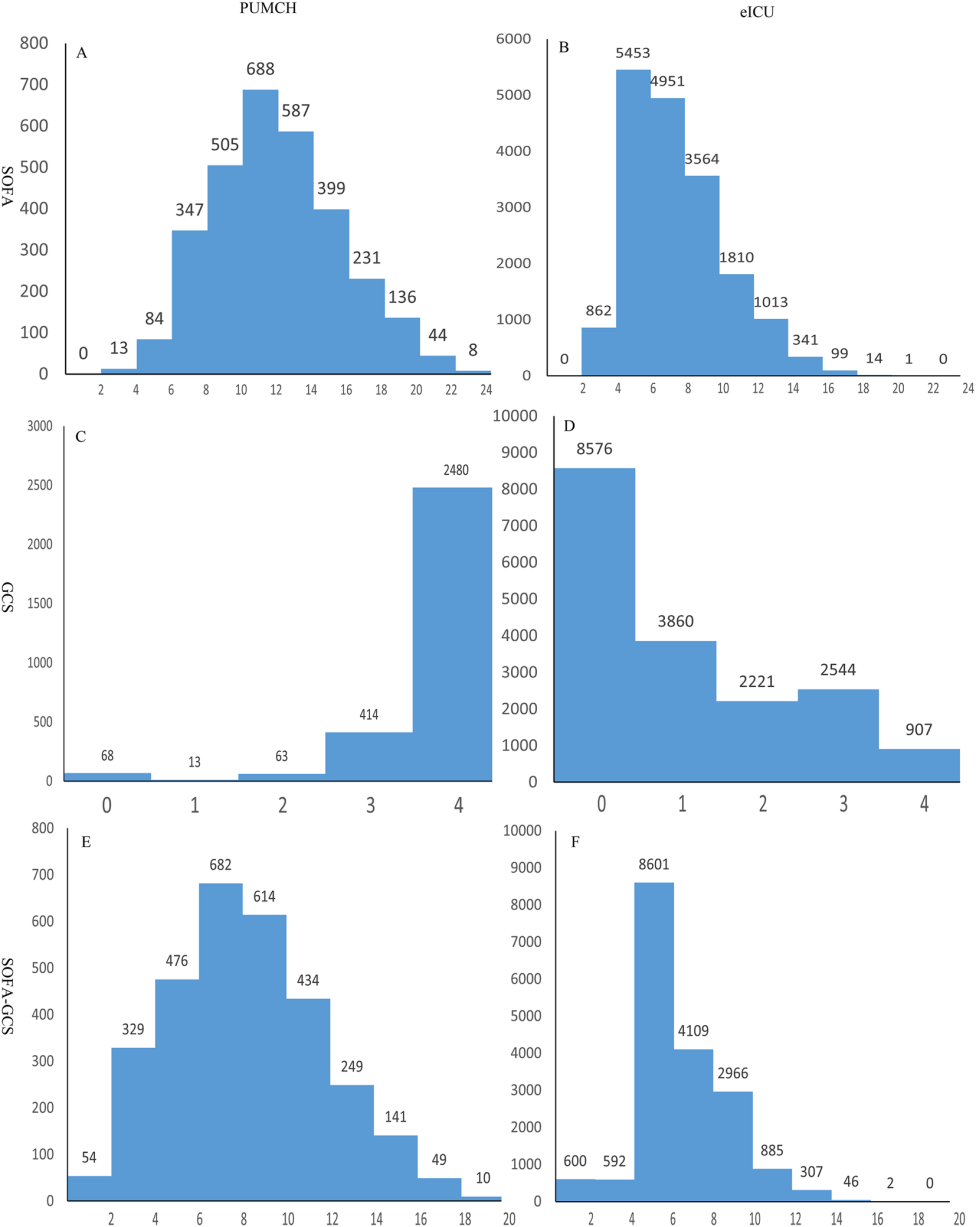
SOFA scores, GCS scores, and SOFA scores without GCS in septic patients in Peking Union Medical College Hospital (PUMCH) and the eICU database

By comparison, we found that the distribution of the included populations was significantly different. SOFA scores, GCS scores, and SOFA scores without GCS in PUMCH were higher than those in the eICU population (Fig. 3). We also found 1769 of the 3048 patients with sepsis were intubated in PUMCH database while 833 of the 18,108 patients with sepsis were intubated in the eICU database.

**Fig. 4:**
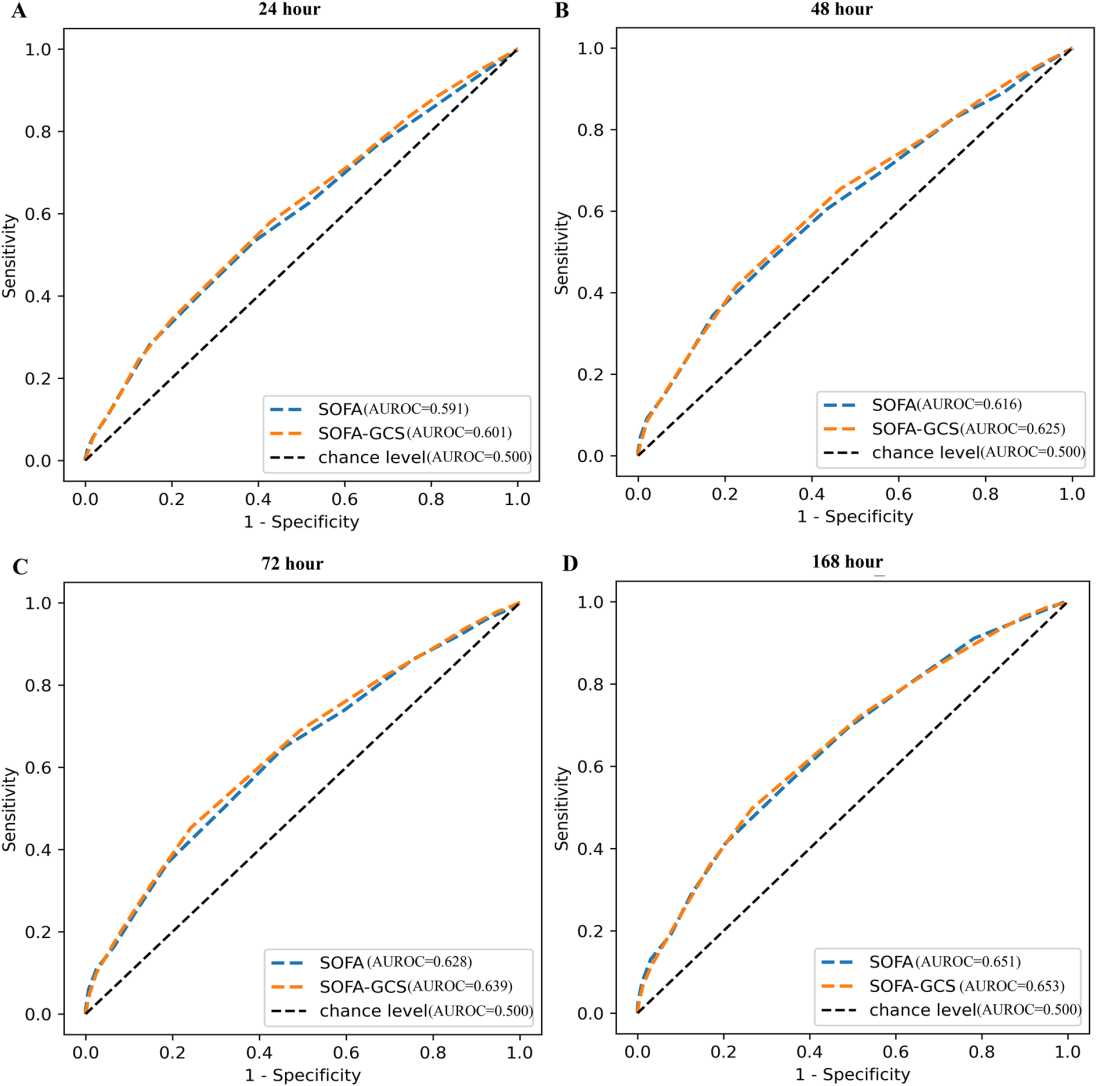
Predictive efficiency of SOFA with or without GCS in septic patients with respiratory system ≥ 3 points in SOFA in the eICU database. **A** p (SOFA vs SOFA-GCS) 0.148, **B** p (SOFA vs SOFA-GCS) 0.178, **C** p (SOFA vs SOFA-GCS) 0.132, **D** p (SOFA vs SOFA-GCS) 0.790

Considering that a considerable number of sepsis patients admitted to various ICUs of PUMCH were intubated and transferred after the patients developed consciousness disorders, we narrowed the range of eICU data to the range of respiratory system ≥ 3 points in SOFA for more accurate comparison. A total of 2397 septic patients with respiratory system ≥ 3 points in SOFA in the eICU database were included. All patients in this subgroup were intubated. We observed that in this subgroup of patients with sepsis in the eICU database, although the difference was not statistically significant (P value, 24 h, 0.148, 48 h, 0.178, 72 h, 0.132, 168 h, 0.790), SOFA without GCS (AUROC [95% CI], 24 h, 0.601 [0.576, 0.626], 48 h, 0.625 [0.601, 0.649], 72 h, 0.639 [0.615, 0.663], 168 h, 0.653 [0.629, 0.677]) had a higher predictive efficiency on ICU mortality than SOFA with GCS (AUROC [95% CI], 24 h, 0.591 [0.566, 0.616], 48 h, 0.616 [0.592, 0.640], 72 h, 0.628 [0.604, 0.652], 168 h, 0.651 [0.627, 0.675]) (Fig. 4).

## Discussion

As the most widely used SOFA in the sepsis diagnosis and treatment related scoring system, efforts to further improve and optimize it have never stopped [9–11]. At present, there are many controversies about the application of SOFA in clinical practice, especially the accuracy of the GCS is the lowest in SOFA [12]. In the initial validation, the best guess method based on clinical experience was used in patients receiving sedation [13]. Other studies continued the last GCS recorded prior to endotracheal intubation until a neurological examination could be performed in patients without sedation. If no value is recorded before intubation, a score of 15 is assumed [14]. However, the timing of intubation is critically dependent on the judgment of the clinician, and a considerable proportion of intubation is due to the patient's impaired consciousness [15]. The central nervous system is an important organ involved in sepsis, and the incidence of sepsis associated encephalopathy is as high as 70%, and its function is constantly and dynamically changing during the course of sepsis [16, 17], a simple approach of best guess or continuing pre-intubation recording may not be appropriate. Central nervous system function is heavily dependent on the normal function of other organs, and there are varying degrees of impaired consciousness in shock, hypoxia [18], liver failure [19], and renal failure [20], so placing central nervous system on the same level as other organs may bias the scoring results in assessment of the severity of sepsis.

With the rise of big data analysis and artificial intelligence technology, the method of machine learning has been widely studied and applied in sepsis patients with massive monitoring data [21–23]. The above situation puts forward higher requirements for the wide application of automatic data collection systems in clinical practice, and the lack of GCS scores as subjective scores often affects the automatic generation of SOFA scores [24], so the search for a more objective SOFA scoring system has become an urgent problem to be solved in clinical practice.

From our study, the use of SOFA without GCS did not affect its predictive efficiency for ICU mortality of sepsis. Even the data from PUMCH showed that SOFA without GCS was significantly better in predictive efficiency, and the difference was statistically significant. In the eICU database, we also observed the same phenomenon in sepsis patients with respiratory system ≥ 3 points in SOFA. Therefore, it is reasonable to believe that it is realistic and feasible to discontinue the routine GCS for SOFA in patients with a respiratory system ≥ 3 points.

There are several limitations to this study. First, similar to digestive system, impaired central nervous function is an important aspect of multi-organ dysfunction, but there are still lack of biomarkers that can represent the function of this system. Simply eliminating the assessment of central nervous function is not the best choice for organ function assessment, and seeking objective indicators to represent its function is a problem that needs to be solved in future research. Second, this was an observational, retrospective study and therefore, prone to selection bias. Third, there are multiple outcome indicators in sepsis, such as mortality, survival time, duration of mechanical ventilation, and length of ICU stay. This study only analyzed ICU mortality [25], which may bias the results. Third, in studies of mortality, 28-day mortality [26, 27] or 30-day mortality [28] may be more objective options, and only ICU mortality [27] was analyzed in this study due to constraints.

## Conclusion

In severe sepsis, it is realistic and feasible to discontinue the routine GCS for SOFA in patients with a respiratory system ≥ 3 points, and even better predict ICU mortality.

### Supplementary Information

**Additional file 1. **STROBE Statement—Checklist of items that should be included in reports of cross-sectional studies.

**Additional file 2. **Patient inclusion and exclusion criteria.

**Additional file 3: Table S1.** Basic information of patients with sepsis in eICU database.

## Data Availability

The datasets supporting the conclusions of this article are included within the article and Additional files.

## Abbreviations

SOFA: Sequential organ failure assessment
GCS: Glasgow Coma Score
PUMCH: Peking Union Medical College Hospital
