# Supplementary figures and images for: SOFA in sepsis: with or without GCS
Source: Eur J Med Res. 2024 May 24;29:296. doi: 10.1186/s40001-024-01849-w (PMC11127461; doi:10.1186/s40001-024-01849-w)

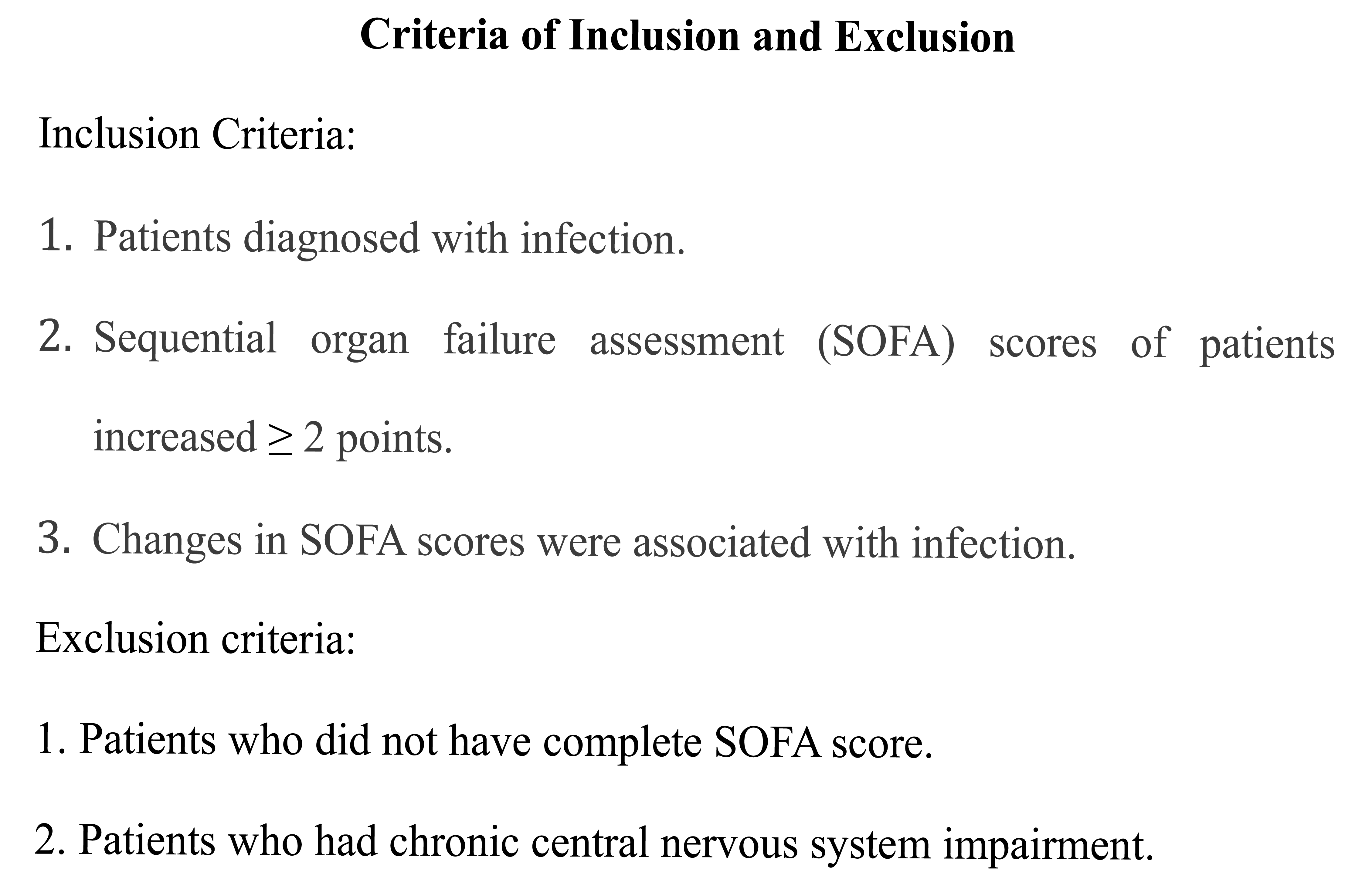

Supplement: Supplementary file 2 — Additional file 2. Patient inclusion and exclusion criteria. [file 40001_2024_1849_MOESM2_ESM.tif]
