# Supplementary material for: SOFA in sepsis: with or without GCS
Source: Eur J Med Res. 2024 May 24;29:296. doi: 10.1186/s40001-024-01849-w (PMC11127461; doi:10.1186/s40001-024-01849-w)
Supplement: Supplementary file 3 — Additional file 3: Table S1. Basic information of patients with sepsis in eICU database. [file 40001_2024_1849_MOESM3_ESM.docx]

**Table S1. Basic information of patients with sepsis in eICU database.**

|  | Patients (n) | Patients died in ICU (n) | ICU mortality (%) |
| --- | --- | --- | --- |
| Total | 18108 | 3091 | 17.07 |
| Female | 8745 | 1500 | 17.15 |
| Age (year) |  |  |  |
| <18 | 8 | 0 | 0 |
| [18, 40) | 1198 | 92 | 7.68 |
| [40, 60) | 4296 | 564 | 13.13 |
| [60, 80) | 8380 | 1501 | 17.91 |
| ≥80 | 4226 | 934 | 22.10 |
| Septic shock | 3890 | 1045 | 26.86 |
| Chronic cardiovascular disease | 2191 | 391 | 17.85 |
| Chronic respiratory diseases | 1380 | 236 | 17.10 |
| Cirrhosis | 318 | 109 | 34.28 |
| Uremia | 967 | 212 | 21.92 |
| Diabetes mellitus | 2477 | 377 | 15.22 |
| Hematological cancer | 374 | 124 | 33.16 |
| Nonhematological cancer | 733 | 191 | 26.06 |
